# Rinne test: does the tuning fork position affect the sound amplitude at the ear?

**DOI:** 10.1186/s40463-016-0133-7

**Published:** 2016-03-24

**Authors:** Oleksandr Butskiy, Denny Ng, Murray Hodgson, Desmond A. Nunez

**Affiliations:** Division of Otolaryngology – Head and Neck Surgery, Vancouver General Hospital, Vancouver, BC Canada; Department of Mechanical Engineering, University of British Columbia, Vancouver, BC Canada; Department of Surgery, University of British Columbia, Vancouver, BC Canada; School of Population and Public Health, University of British Columbia, Vancouver, BC Canada; Gordon & Leslie Diamond Health Care Centre, 4th. Fl. 4299B-2775 Laurel Street, Vancouver, BC V5Z 1M9 Canada

**Keywords:** Tuning fork, Physical examination, Rinne test

## Abstract

**Background:**

Guidelines and text-book descriptions of the Rinne test advise orienting the tuning fork tines in parallel with the longitudinal axis of the external auditory canal (EAC), presumably to maximise the amplitude of the air conducted sound signal at the ear. Whether the orientation of the tuning fork tines affects the amplitude of the sound signal at the ear in clinical practice has not been previously reported. The present study had two goals: determine if (1) there is clinician variability in tuning fork placement when presenting the air-conduction stimulus during the Rinne test; (2) the orientation of the tuning fork tines, parallel versus perpendicular to the EAC, affects the sound amplitude at the ear.

**Methods:**

To assess the variability in performing the Rinne test, the Canadian Society of Otolaryngology – Head and Neck Surgery members were surveyed. The amplitudes of the sound delivered to the tympanic membrane with the activated tuning fork tines held in parallel, and perpendicular to, the longitudinal axis of the EAC were measured using a Knowles Electronics Mannequin for Acoustic Research (KEMAR) with the microphone of a sound level meter inserted in the pinna insert.

**Results:**

47.4 and 44.8 % of 116 survey responders reported placing the fork parallel and perpendicular to the EAC respectively. The sound intensity (sound-pressure level) recorded at the tympanic membrane with the 512 Hz tuning fork tines in parallel with as opposed to perpendicular to the EAC was louder by 2.5 dB (95 % CI: 1.35, 3.65 dB; *p* < 0.0001) for the fundamental frequency (512 Hz), and by 4.94 dB (95 % CI: 3.10, 6.78 dB; *p* < 0.0001) and 3.70 dB (95 % CI: 1.62, 5.78 dB; *p* = .001) for the two harmonic (non-fundamental) frequencies (1 and 3.15 kHz), respectively. The 256 Hz tuning fork in parallel with the EAC as opposed to perpendicular to was louder by 0.83 dB (95 % CI: −0.26, 1.93 dB; *p* = 0.14) for the fundamental frequency (256 Hz), and by 4.28 dB (95 % CI: 2.65, 5.90 dB; *p* < 0.001) and 1.93 dB (95 % CI: 0.26, 3.61 dB; *p* = .02) for the two harmonic frequencies (500 and 4 kHz) respectively.

**Conclusions:**

Clinicians vary in their orientation of the tuning fork tines in relation to the EAC when performing the Rinne test. Placement of the tuning fork tines in parallel as opposed to perpendicular to the EAC results in a higher sound amplitude at the level of the tympanic membrane.

## Background

Historically, up to 20 tuning fork tests were used in the diagnosis of hearing loss [1]. Anecdotally only two tests, Webber and Rinne, continue to be routinely taught in medical schools and used clinically by otologists and primary care physicians. The Rinne test is recommended as part of an otological physical exam to detect conductive hearing loss [2]. In patients with otosclerosis, the Rinne test is used to determine stapes surgery candidacy [3]. Olotaryngologists have advocated for further study of the sources of variation in performing the Rinne test given its widespread clinical use [4].

Audiology society recommendations [5] instructions aimed at medical student and non-specialist on performing the Rinne test in general and otolaryngology textbooks [6], instructions intended for otolaryngology residents in speciality textbooks [7], and peer reviewed publications [4, 8] all describe placing the vibrating tuning fork tines in parallel with the longitudinal axis of external auditory canal (or parallel to the frontal plane of the skull). In comparison to perpendicular placement of the tines, placement of the tines parallel to the ear canal is thought to result in higher sound intensities (i.e., sound pressure levels) at the patient’s eardrum [5].

Mathematical calculations and sound field recordings conclude that a higher amplitude sound is delivered to the ear when the fork is placed parallel to as opposed to perpendicular to the EAC [9, 10]. These lines of evidence show a 5 dB difference in the sound intensity produced by the two different positions of the tuning fork [10]. However, there are several known tuning fork vibration modes, and these mathematical models and experimental studies have only tested the individual vibration modes. A tuning fork activated by a physician likely produces a sound that is a product of at least seven known vibration modes [11]. The sound intensities of a tuning fork placed parallel to and perpendicular to the EAC during the Rinne test have not been compared before.

The present study had two goals: To determine if (1) Canadian otolaryngologists demonstrate variability in performance of the Rinne test, specifically focusing on the tuning fork placement during air conduction testing; (2) orientation of the tuning fork tines, parallel to as compared to perpendicular to the EAC, affects the amplitude of sound (at fundamental and harmonic frequencies) at the level of the tympanic membrane.

## Methods

To assess the variability in performance of the Rinne test amongst Canadian otolaryngologists, we conducted an e-mail survey through the Canadian Society of Otolaryngology – Head and Neck Surgery member e-mail list. Prior to conducting the survey, ethics approval from our institution was sought, but was deemed unnecessary by the research ethics board. The survey was e-mailed out once to the member list on April 22nd, 2015 and the results were collected until June 2nd, 2015. The survey consisted of four multiple-choice questions and a comment section.

**Fig. 1:**
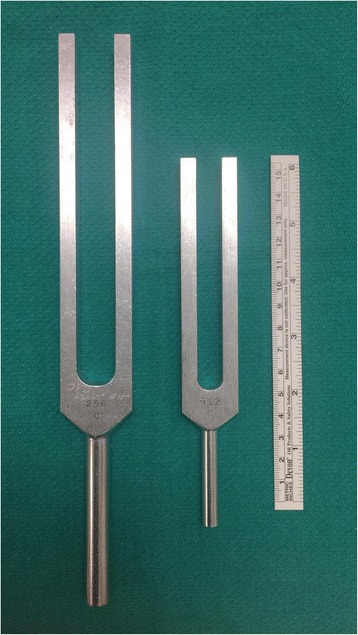
256 Hz (*left*) and 512 Hz (*right*) tuning forks used in the experiment

An experimental simulation of the air conduction component of the Rinne test was used to measure the sound intensity at the level of the tympanic membrane for both parallel and perpendicular positions of the tuning fork. Two aluminum tuning forks (512 Hz and 256 Hz) of the same design were used in the experiment (Fig. 1).

**Fig. 2:**
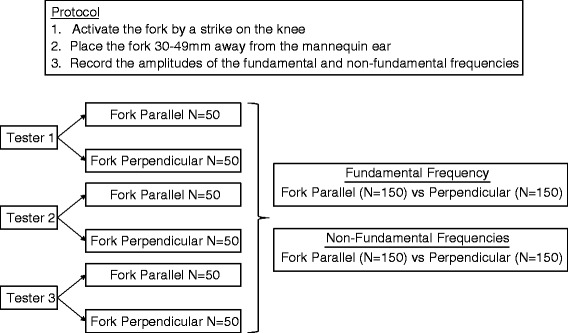
The experimental design

**Fig. 3:**
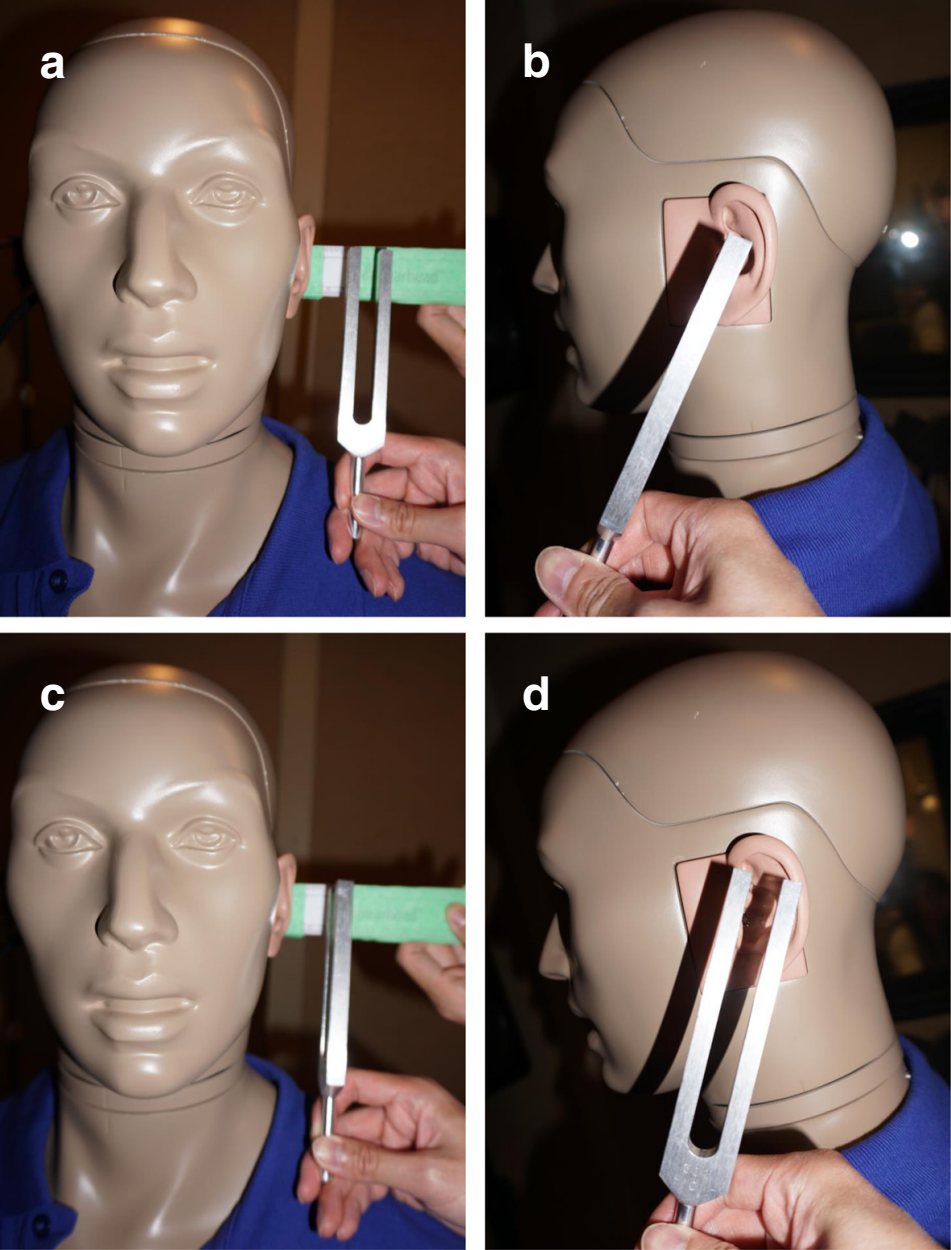
Simulation of the Rinne Test: placement of the 512 Hz tuning fork parallel (**a**, **b**) and perpendicular (**c**, **d**) to the ear canal

The experimental design is summarized in Fig. 2. The protocol for tuning fork activation and placement was based on the most common responses from the email survey. One of the testers was blinded to the study question. A visual reference was used to train the testers to consistently place the edge of the vibrating tuning fork 30–49 mm lateral to the ear canal (Fig. 3a, c). In addition, the testers were trained to align the middle of tuning fork with the EAC viewed in the coronal plane (Fig. 3b, d). To ensure consistent tuning fork placement throughout the experiment, the placement of the tuning fork was re-checked using a visual reference after each of 50 consecutive activations.

The sound intensities produced by the tuning fork during individual activations were recorded with a RION NA-28 Sound Level Meter (RION Co., Ltd., Tokyo, Japan) with its microphone inserted into the EAC hole in the pinna insert of a KEMAR Manikin Type 45BA (G.R.A.S. Sound & Vibration, Holte, Denmark). The sound spectra of the tuning forks were measured in 1/3 octave bands. Each measurement was triggered when the 1/3 octave band of interest (256 or 512 Hz) exceeded 70 dB. This helped reduce variability associated with different excitations, and positionings, of the tuning fork. Once triggered, the measurements were taken over 3 s and averaged.

An independent-samples *t*-test was used to compare the parallel and perpendicular placements of the tuning fork with respect to the measured amplitudes of the fundamental frequencies (512 and 256 Hz) and dominant harmonic frequencies. The dominant harmonic frequencies were identified by visual inspection of the averaged sound spectrum of each tuning fork activation.

## Results

### (1) Email survey

Out of 512 active members of the CSO-HNS, 116 physicians responded to the survey for a response rate of 23 % (Tables 1, 2, 3, and 4). 113 responders reported practicing in Canada. The highest proportion of the responders reported using a 512 Hz tuning fork (73 %; 85 responders), activating the fork by a strike on the knee (45.7 %; 55 responders), and holding the fork 3 to 4 cm away from the ear (44.8 %; 52 responders). 55 (47.4 %) of the surveyed physicians reported placing the fork parallel, and 52 (44.8 %) reported placing the fork perpendicular to the ear canal.

**Table 1:** Canadian Society of Otolaryngology - Head and Neck Surgery e-mail survey results (116 Responders)

What Frequency of tuning fork do you use to administer the Rinne test?		
256 Hz	512 Hz	Other
16	83	17

**Table 2:** Canadian Society of Otolaryngology - Head and Neck Surgery e-mail survey results (116 Responders)

How do you mostly activate the tuning fork for the Rinne test?				
Elbow Strike	Knee Strike	Striking a soft coated surface	Finger Pinch	Other
38	53	10	3	12

**Table 3:** Canadian Society of Otolaryngology - Head and Neck Surgery e-mail survey results (116 Responders)

How far from the ear do you hold the tuning fork?					
12 cm	3–4 cm	5–6 cm	7–8 cm	9–10+ cm	other
45	52	11	4	0	4

**Table 4:** Canadian Society of Otolaryngology - Head and Neck Surgery e-mail survey results (116 Responders)

During the Rinne test, are the tines of the fork parallel or perpendicular to the auditory canal?		
Parallel	Perpendicular	Other
55	52	9

### (2) Simulation of the Rinne air conduction testing

**Fig. 4:**
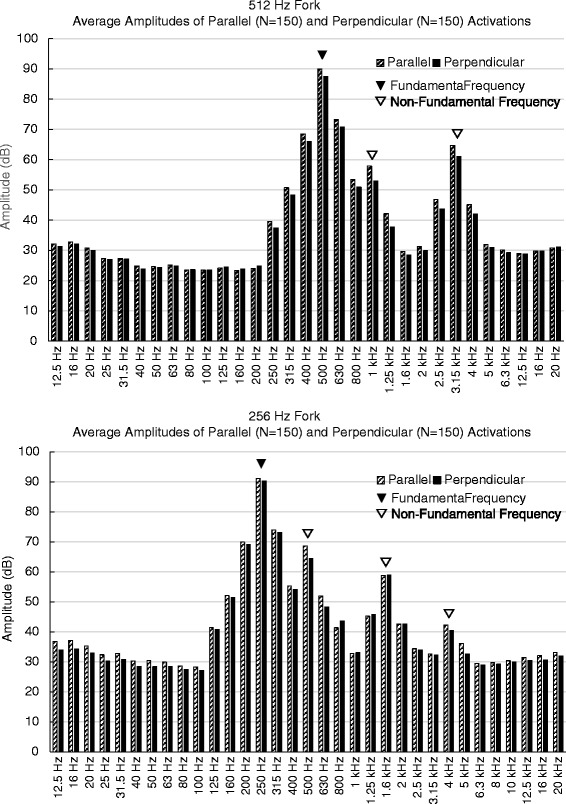
Average amplitudes obtained by activating 512 and 256 Hz tuning forks in parallel (*shaded bars*) and perpendicular (*solid bars*). The fundamental frequencies are marked with solid arrow heads; the main non-fundamental frequencies are marked with empty arrow heads

The average amplitudes of the sound spectra produced by 512 and 256 Hz tuning forks placed parallel and perpendicular to the ear canal are presented in Fig. 4. Visual inspection of the sound spectra of each tuning fork identified two dominant harmonic frequencies for the 512 Hz tuning fork (1 and 3.15 kHz) and three dominant harmonic frequencies for the 256 Hz tuning fork (500 Hz, 1.6, and 4 kHz).

The statistical comparison of parallel and perpendicular placements of the 512 and 256 Hz tuning forks with respect to the amplitude of the fundamental frequencies and dominant harmonic frequencies are summarized in Tables 5 and 6. The sound intensity recorded at the tympanic membrane with the 512 Hz tuning fork tines in parallel with as opposed to perpendicular to the EAC was louder by 2.5 dB (95 % CI: 1.35, 3.65 dB; *p* < 0.0001) for the fundamental frequency (512 Hz), and by 4.94 dB (95 % CI: 3.10, 6.78 dB; *p* < 0.0001) and 3.70 dB (95 % CI: 1.62, 5.78 dB; *p* = .001) for the two harmonic frequencies (1 and 3.15 kHz) respectively (Table 5). The 256 Hz tuning fork in parallel with the EAC as opposed to perpendicular to was louder by 0.83 dB (95 % CI: −0.26, 1.93 dB; *p* = 0.14) for the fundamental frequency (256 Hz), and by 4.28 dB (95 % CI: 2.65, 5.90 dB; *p* < 0.001) and 1.93 dB (95 % CI: 0.26, 3.61 dB; *p* = .02) for the two harmonic frequencies (500 and 4 kHz) respectively (Table 6). For the 1.6 kHz harmonic frequency of the 256 Hz tuning fork, the perpendicular placement of the tuning fork was louder than parallel placement of the tuning fork by 0.11 dB (95 % CI: −1.58, 1.8 dB; *p* = 0.89).

**Table 5:** Sound amplitudes produced by parallel and perpendicular placement of 512Hz fork at the selected frequencies

Frequency Measured	Mean Amplitude (±SD)		Mean Difference (95 % CI)	*p*-value
	Parallel (*N* = 150)	Perpendicular (*N* =150)		
500 Hz	90.04 dB (±4.46 dB)	87.53 dB (±5.63 dB)	2.50 dB (±1.15 dB)	<0.0001
1 kHz	57.86 dB (±7.64 dB)	52.92 dB (±8.50 dB)	4.94 dB (±1.84 dB)	<0.0001
3.15 kHz	64.76 dB (±7.32 dB)	61.05 dB (±10.69 dB)	3.70 dB (±2.08 dB)	.001

*SD* Standard Deviation, *CI* Confidence Interval

**Table 6:** Sound amplitudes produces by parallel and perpendicular placement of 256Hz fork at the selected frequencies

Frequency Measured	Mean Amplitude (±SD)		Mean Difference (95 % CI)	p-value
	Parallel (*N* = 150)	Perpendicular (*N* =150)		
250 Hz	91.14 dB (±4.06 dB)	90.30 dB (±5.47 dB)	0.83 dB (±1.09 dB)	.14
500 kHz	68.67 dB (±7.30 dB)	64.39 dB (±6.99 dB)	4.28 dB (±1.62 dB)	<0.001
1.6 kHz	58.80 dB (±7.13 dB)	58.92 dB (±7.73 dB)	−0.11 dB (±1.69 dB)	.89
4 kHz	42.35 dB (±8.52 dB)	40.42 dB (±6.05 dB)	1.93 dB (±1.67 dB)	.02

*SD* Standard Deviation, *CI* Confidence Interval

## Discussion

The results of the e-mail survey show that despite the use of the Rinne test by the majority of the responding otolaryngologists, the air conduction testing techniques in use are not uniform. The survey suggests that the majority of Canadian otolaryngologists prefer the 512 Hz tuning fork, activate the fork by the strike of the knee, and place the fork approximately 3 to 4 cm away from the ear canal when testing air conduction. Despite the traditional teaching on the placement of the tuning fork tines during air conduction testing, the results of the survey show a roughly equal use of parallel and perpendicular tuning fork placement amongst the responders. Whilst some of the responders did not understand what was meant by parallel and perpendicular placement of the fork, these findings suggest that Canadian Otolaryngologists vary in their orientation of the tuning fork tines.

The results of the survey should be interpreted with caution. Only a limited number of physicians responded to the survey (23 % response rate). Furthermore, the question design only allowed for a limited number of responses. Therefore, the complete variability in air conduction testing by Canadian otolaryngologists has likely not been captured by the survey. Despite these limitations, the survey provided useful information for designing the experimental part of the study.

To our knowledge, the sound spectra for the 512 and 256 Hz tuning forks activated in clinical practice for the purposes of the Rinne test have not been documented previously. The sound spectra (Fig. 4) and the knowledge of the dominant harmonic frequencies are valuable for interpreting Rinne test results for patients with different levels of hearing loss across the frequency spectrum.

The experimental findings support the traditional teaching that parallel placement of tuning fork tines with respect to the EAC produces higher sound amplitude at the level of the tympanic membrane than perpendicular placement of the tines. For the 512 Hz tuning fork, the difference between the two positions of the tuning fork was measured to be 2.5 dB for the fundamental frequency. This is less than the 5 dB difference predicted by the mathematical models [10]. The smaller than expected difference could be due to the complex interactions of the tuning fork vibration modes not accounted for by the mathematical models. Alternatively, this smaller difference could be explained by the inherent variability in activations of the tuning fork by a strike on the knee.

**Fig. 5:**
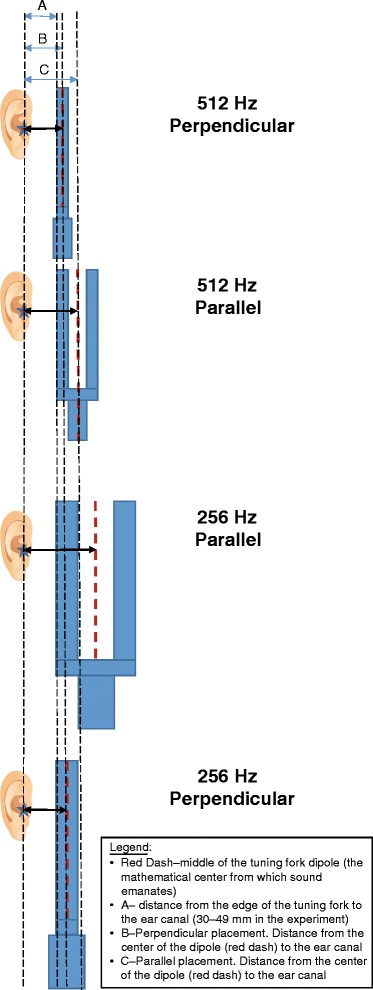
The influence of tuning fork size on the distance from the centre of the tuning fork dipole to the ear canal. Parallel orientation produces a louder sound and when this is coupled with placement of the vibrating dipole closer to the ear canal in the smaller 512 Hz tuning fork the effect is most marked

The measured 0.83 dB fundamental frequency amplitude difference between the parallel and perpendicular placement of the 256 Hz tuning fork was smaller than the 2.5 dB difference measured for the 512 Hz tuning fork. Even though the amplitude for the parallel placement of the 256 Hz tuning fork was again greater than for the perpendicular placement, this difference did not reach statistical significance. The explanation for the lack of statistical significance likely lies in the difference of geometry between the 512 and 256 Hz fork. Due to the need to keep the design of the 512 and 256 Hz tuning forks consistent, the 256 Hz tuning fork was larger than the 512 Hz tuning fork (Fig. 1). Given, its larger dimensions, the difference in the amplitude between the parallel and perpendicular placement of the 256 Hz tuning fork was likely negated by the wider vibration field of the larger tines: when testing the parallel position of the tuning fork, placing the edge of the 256Hz fork 30 to 49 cm away from the EAC positions the centre of the tuning fork further away from the EAC as compared to the same placement of the smaller 512Hz tuning fork (Fig. 5). We tested this explanation by performing a separate experiment with a different design of the 256 Hz tuning fork, where the dimensions of the 256 Hz fork were similar to the 512 Hz fork. In this separate experiment, not presented in this report, a statistically significant difference of 3.7 dB in favour of the parallel placement of the tuning fork was found.

Loudness perception is a complicated psychoacoustic phenomenon influenced not only by the amplitude but also by the frequency of the sound, its spectral distribution, its duration and time structure, and by its overall acoustic environment [12]. Assuming that all other variables influencing the perception of loudness are kept constant, a normal hearing individual should be able to discriminate a difference in amplitude as small as 1.5 dB [13, 14]. The amplitude resolution of 1.5 dB is preserved in hearing-impaired patients with most types of conductive and sensorineural hearing loss. The only apparent exception is the lower amplitude resolution seen in patients with acoustic neuroma (4.5 dB) [13, 14]. These facts suggest that the amplitude difference between in parallel and perpendicular to the EEC tuning fork placement observed in this study can be perceived by most patients undergoing the Rinne test. Thus, the position of the tuning fork with respect to the EAC during the Rinne test represents a significant variable that can potentially influence the sensitivity and specificity of the test. Further investigation is needed to test whether the position of the tuning fork during the Rinne test affects the its results in patients with hearing loss.

## Conclusions

Despite widespread use of the Rinne test by Canadian otolaryngologists, the Rinne test techniques practiced are non-uniform. Orientation of the tuning fork tines with respect to the EAC during air conduction testing is an important source of variation in performing the Rinne test. Placement of the tuning fork tines in parallel as opposed to perpendicular to the ear canal produces a sound of higher amplitude at the level of the tympanic membrane. Physicians are encouraged to pay attention to the orientation of tuning fork’s tines with respect to the long axis of the EAC when testing air conduction during the Rinne test.

## Abbreviations

EAC: external ear canal
